# Correction: Recombinant Yellow Fever Viruses Elicit CD8^+^ T Cell Responses and Protective Immunity against *Trypanosoma cruzi*

**DOI:** 10.1371/annotation/39b41d98-b117-41cf-b5de-b8486a67b1cd

**Published:** 2013-11-08

**Authors:** Raquel Tayar Nogueira, Alanderson Rocha Nogueira, Mirian Claudia Souza Pereira, Maurício Martins Rodrigues, Patrícia Cristina da Costa Neves, Ricardo Galler, Myrna Cristina Bonaldo

Most of the in-text references in this article are missing from the body of the text. A corrected version with references in place can be found here:

Download corrected item

