# Supplementary material for: Correction: Recombinant Yellow Fever Viruses Elicit CD8+ T Cell Responses and Protective Immunity against Trypanosoma cruzi
Source: PLoS One. 2013 Nov 8;8(11):10.1371/annotation/39b41d98-b117-41cf-b5de-b8486a67b1cd. doi: 10.1371/annotation/39b41d98-b117-41cf-b5de-b8486a67b1cd (PMC5752289; doi:10.1371/annotation/39b41d98-b117-41cf-b5de-b8486a67b1cd)
Supplement: Download corrected item [file 39b41d98-b117-41cf-b5de-b8486a67b1cd.s001.doc]

**Recombinant Yellow Fever Viruses elicit CD8^+^ T cell responses and protective immunity against *Trypanosoma cruzi***

Raquel Tayar Nogueira^1^, Alanderson Rocha Nogueira^3^, Mirian Claudia Souza Pereira^3^, Maurício Martins Rodrigues^4^, Patrícia Cristina da Costa Neves^1,5^, Ricardo Galler^2^ and Myrna Cristina Bonaldo^1§^

Affiliations:

^1^Fundação Oswaldo Cruz, Instituto Oswaldo Cruz, Laboratório de Biologia Molecular de Flavivírus, Rio de Janeiro, Fundação Oswaldo Cruz. Avenida Brasil 4365, Manguinhos, Rio de Janeiro, RJ, Brazil, 21045-900

^2^Fundação Oswaldo Cruz, Instituto de Tecnologia em Imunobiológicos, Rio de Janeiro, Brazil

^3^Fundação Oswaldo Cruz, Instituto Oswaldo Cruz, Laboratório de Ultra-Estrutura Celular, Rio de Janeiro, Brazil

^4^Centro de Terapia Celular e Molecular, Departamento de Microbiologia, Imunologia e Parasitologia, Escola Paulista de Medicina, Universidade Federal de São Paulo, Rua Mirassol 207, 04044-010 São Paulo, SP, Brasil.

^5^Fundação Oswaldo Cruz, Instituto Oswaldo Cruz, Laboratório de Tecnologia Imunológica- Vice Diretoria de Desenvolvimento Tecnológico - Bio-Manguinhos, Rio de Janeiro, Brazil

^§^Corresponding author: mbonaldo@ioc.fiocruz.br

**ABSTRACT**

Chagas’ disease is a major public health problem affecting nearly 10 million in Latin America. Despite several experimental vaccines have shown to be immunogenic and protective in mouse models, there is not a current vaccine being licensed for humans or in clinical trial against *T. cruzi* infection. Towards this goal, we used the backbone of Yellow Fever (YF) 17D virus, one of the most effective and well-established human vaccines, to express an immunogenic fragment derived from *T. cruzi* Amastigote Surface Protein 2 (ASP-2). The cDNA sequence of an ASP-2 fragment was inserted between E and NS1 genes of YF 17D virus through the construction of a recombinant heterologous cassette. The replication ability and genetic stability of recombinant YF virus (YF17D/ENS1/Tc) was confirmed for at least six passages in Vero cells. Immunogenicity studies showed that YF17D/ENS1/Tc virus elicited neutralizing antibodies and gamma interferon (IFN-γ) producing-cells against the YF virus. Also, it was able to prime a CD8^+^ T cell directed against the transgenic *T. cruzi* epitope (TEWETGQI) which expanded significantly as measured by T cell-specific production of IFN-γ before and after *T. cruzi* challenge. However, most important for the purposes of vaccine development was the fact that a more efficient protective response could be seen in mice challenged after vaccination with the YF viral formulation consisting of YF17D/ENS1/Tc and a YF17D recombinant virus expressing the TEWETGQI epitope at the NS2B-3 junction. The superior protective immunity observed might be due to an earlier priming of epitope-specific IFN-γ-producing T CD8^+^ cells induced by vaccination with this viral formulation. Our results suggest that the use of viral formulations consisting of a mixture of recombinant YF 17D viruses may be a promising strategy to elicit protective immune responses against pathogens, in general.

**INTRODUCTION**

Chagas’ disease, caused by *Trypanosoma cruzi* is considered a neglected infectious disease, with an estimated 7 to 10 million cases in Latin America and about 10,000 to 14,000 deaths annually . The current state of globalization of Chagas’ disease due to high immigration to non-endemic countries and also the high economic impact in lost productivity, has highlighted this emerging disease as a major public health challenge. This scenario has increased government efforts in trying to prevent the spread of *T. cruzi* and still has encouraged advances in the treatment of the disease and development of preventive and therapeutic vaccines ,. Many recombinant proteins, DNA, viral vectors and heterologous prime-boost regimens of vaccination suggest that it is feasible to control *T. cruzi* infection by vaccination (reviewed by ). Despite these promising results not a single candidate vaccine has been tested in humans. This is still an intense field of investigation and could bring economic benefits .

It has been a consensus that a Th1 response with the stimulation of CD8^+^ T cell controls *T. cruzi* infection in murine models and reduces the severity of the disease in humans . The production of pro-inflammatory cytokines such as gamma-interferon (IFN-γ) and tumor necrosis factor-alpha (TNF-α) by CD8^+^ T cells is essential to a protective response against *T. cruzi* given that they stimulate the production of nitric oxide (NO) by macrophages which is directly involved in the reduction of *T. cruzi* parasitemia and *in vitro* parasite killing . In addition, it was previously shown that a delay in the CD8^+^ T cells expansion (and IFN-γ production) after a *T. cruzi* challenge may be an efficient mechanism to launch the parasite infection in naïve mice

Different strategies have been employed in the search of an effective vaccine against *T. cruzi*, including genetically attenuated parasites , recombinant proteins , viral vectors and DNA vaccines . Thus, many antigens of *T. cruzi* as the *trans*-sialidases (TS), one of the largest protein families of the parasite , cruzipain , paraflagellar rod protein-2 , GP82 , trypomastigotes excretory-secretory antigens or kinetoplastid protein KMP11 has been the focus of vaccine development. Among them, the amastigote surface protein (ASP-2) has displayed enhanced antigenicity in several vaccine experimental models .

Previous studies have shown that immunization with recombinant proteins or genes based on the amastigote surface protein-2 (ASP-2) , as well as heterologous prime-boost, using DNA and recombinant viral vectors expressing ASP-2 antigens conferred encouraging protective immunity against *T. cruzi*. These and other studies provided evidence that CD8^+^ and CD4^+^ Th1 cells are particularly important to the development of acquired immunity against experimental infection in mice . These data were obtained in a *T. cruzi* highly susceptible mouse strain (A/J) that totally succumb with a relatively small dose of the Y strain of the *T. cruzi* parasite after a short period of time (less than 30 days after infection).

The Yellow Fever vaccine virus (YF 17D) is a well-established human vaccine that has proven to set off a polyvalent innate immune response resulting in life-long immunity that includes a robust neutralizing antibody response that may persist for up to 40 years after vaccination , , a mixed CD4^+^ T helper (Th1 and Th2) cell profile and a potent cytotoxic CD8^+^ T cell response . An important aspect of the YF 17D vaccine is its capacity to induce specific CD8^+^ T cells early after vaccination (5 days) in humans or in mice . Moreover, the production of the soluble mediator IFN-γ, which plays an essential role in YF infection , is also initiated 5 to 7 days after YF vaccination . Therefore we expect that YF 17D virus could be the vector of choice to elicit the appropriate immune response since it is well known that a rapid CD8^+^ T cell induction (and IFN-γ production) are advantageous features against *T. cruzi* infection in mice and humans .

Based on these data following vaccination of A/J mouse model, we attempted to build on this strategy by generating recombinant YF 17D viruses that express CD4^+^ and CD8^+^ T cells epitopes derived from the ASP-2. We have recently reported the characterization of two immunogenic recombinant YF 17D viruses that expressed the immunodominant CD8^+^ T cell, TEWETGQI epitope of ASP-2 in the envelope protein (E) and in the intergenic region of NS2B and NS3 protease . Immunization of A/J mice with the recombinant YF 17D viruses induced a TEWETGQI-specific CD8^+^ T cell response and delayed mouse mortality after a *T. cruzi* challenge.

In this work, we approached the expression of a larger fragment of ASP-2 (ASP-2_261-380_) , inserting it between the E (Envelope) and the non-structural NS1 protein coding genes of YF 17D virus by means of a previously established methodology ,. We studied the protective immunogenicity of this recombinant ASP-2_261-380_ YF 17D virus in mice administrating it alone or combined with the YF 17D virus expressing the ASP-2 TEWETGQI epitope between NS2B and NS3 proteins. In the latter, we would like to verify whether the simultaneously immunization recombinant YF 17D viruses, which may present antigens through different cell compartments (at the endoplasmatic reticulum or in the cytosol, respectively), and most likely leading to distinct pathways of immune-presentation, could elicit a more protective immune response against *T. cruzi*. In this regard, our results demonstrate for the first time that the co-administration of recombinant yellow fever viruses can elicit enhanced protection of the animals against *T. cruzi* challenge with decrease in the rates of mortality and parasitemia representing thus a promising strategy to develop a vaccine for Chagas’ disease.

**MATERIALS AND METHODS**

Mice and Parasites

Female 4 to 6 week-old A/J mice in this study were obtained from CECAL, Fiocruz (Rio de Janeiro, Brazil) or purchased from CEMIB/UNICAMP. This study was carried out in strict accordance with the recommendations in the Guide for the National Council for Control of Animal Experimentation (CONCEA). The protocol was approved by the Committee on the Ethics of Animal Experiments (CEUA) of Oswaldo Cruz Foundation (Permit Number: L-0032). Accordingly, all surgery was performed under anesthesia, and all efforts were made to minimize suffering. Animals were twice monitored daily (morning and evening) by a technician responsible for evaluating the appearance of clinical signs such as changes in behavior (low activity, abnormal locomotion, unusual aggressiveness and continuous licking of body parts), piloerection or frequency of feeding (decreasing of food or water consumption). Occurrence of some of these symptoms were evaluated as signs of animal distress and suffering and were critical to the ultimate decision of euthanasia for humane reasons. Mice were human sacrificed by intraperitoneal injection of sodium thiopental (200 mg/ kg). After allowing sufficient time for the animal to lose consciousness, we performed exsanguination and spleen collection of each animal.

Bloodstream trypomastigote forms of *T. cruzi*, Y strain, were obtained from *T. cruzi*-infected Swiss mice at the peak of the parasitemia. Briefly, male mice (18-20g) were inoculated intraperitoneally with 3x10^5^ trypomastigotes. Total blood sample was harvested by heart puncture after 7 days post-infection and the parasites were purified by differential centrifugation

Cell culture and vaccine virus

Vero cells (ATCC) were grown in Earle’s 199 medium supplemented with 5% fetal bovine serum (FBS). As a vaccine control virus, we have utilized the YF sub-strain 17DD (Bio-Manguinhos, Rio de Janeiro, Brazil).

Virus recovery, biological and genetic studies

The recombinant ASP-2_261-380_ cassette was constructed by amplification of ASP-2 gene cloned in the plasmid pIgSPclone9 (GenBank: AY186572.1 . The strategy of the heterologous cassete construction was based on the approach previously described . Initially, the ASP-2_261-380_ fragment of 406 bp was amplified with the positive primer corresponding to 27 nucleotides of the NS1 gene and 20 nucleotides from ASP-2_261-380_ and the negative primer corresponding to the last 27 nucleotides of the ASP-2_261-380_ gene plus the initial 27 nucleotides coding for the amino-terminal domain of the DEN4 E protein stem-anchor region. The second fragment (336 bp) was based on the amplification of the YF cDNA plasmid and was made of 21 nucleotides from the carboxi-terminal of the ASP-2_261-380_ gene followed by the DEN4 stem-anchor region (288 bp) and 27 nucleotides from the amino-terminus of the NS1 gene. Both fragments were mixed in equimolar amounts and reamplified with terminal cassete primers. The resulting fragment of 708 bp was purified with silica-based kit (QIAGEN) and cloned in the pCR-Blunt II TOPO plasmid (*Invitrogen*) using chemically competent *E. coli* DH5-α. The insert was removed by digestion with *Nar* I and *Eag* I and ligated into YF pT3 plasmid. The insertion and its orientation was verified by nucleotide sequencing.

To recover the recombinant YF17D/ENS1/Tc virus we have employed the two infectious-plasmid clone technology, as previously described . After the transfection of viral RNA into Vero cells and the onset of cytopathic effect, the viral supernatant was collected and used to re-infect Vero cell monolayers, generating a second passage viral stock for all subsequent experiments.

Viral proliferation curves were determined by infecting monolayers of Vero cells at MOI of 0.02. Cells were seeded at a density of 62,500 cell/cm^2^ and infected 24 hs later. Samples of the cell culture supernatant were collected at 24-hour intervals post-infection. Viral yields were estimated by plaque titration on Vero cells. The different viral growth peaks were compared using the Mann-Whitney test. (GraphPad Prism 5.03 Program). The differences were only considered significant when *P* < 0.05.

To access the integrity of the heterologous ASP-2 insert, viral RNA samples were obtained and used as a template for cDNA synthesis as previously described . Amplification of the viral genomic E-NS1 region encompassing the heterologous insert was performed using an antisense (YF genome position 2619 – 2639) and sense primer (genome position 1639 – 1659). PCR products were submitted to nucleotide sequencing as described elsewhere . In the genetic stability studies, recombinant viruses were submitted to two independent series of six passages in Vero cells at MOI of 0.02. In the second and the sixth passage, viral RNA was extracted at 72 hs post-infection and submitted to RT/PCR and nucleotidic sequencing.

Detection of YF and *T.cruzi* antigens in Vero cells by immunofluorescence studies.

To detect the expression of YF and ASP-2 antigens in YF virus infected Vero cells immunofluorescence analysis was employed as previously described . As primary antibodies, mouse anti-TEWETGQI or mouse hyperimmune ascitic fluid to YF 17D (ATCC) were used. As secondary antibodies, we used Alexa Fluor 546 goat anti-mouse IgG-Invitrogen or Alexa Fluor 488 goat anti-mouse IgG-Invitrogen, according to the manufacturer’s recomendations. To determine the reactivity to *T.cruzi* amastigotes of sera from vaccinated mouse groups to *T. cruzi* amastigotes, Vero cells were infected during 24 hours with 10^6^ *T. cruzi* trypomastigotes (Y strain) in a 10:1 (parasite/cell) rate. After this time, parasites were removed with PBS washing and infected-Vero cells were incubated for an additional period of 72 hours. Cell monolayer was fixed and washed with PBS containing 0.5% of Triton X-100 and permeabilized followed by incubation with PBS 4% BSA. *T. cruzi* amastigotes infected-cells were then incubated with sera of mice immunized with YF17/ENS1/Tc. Secondary fluorescein isothiocyanate (FITC)-conjugated anti-mouse antibodies were used. Both preparations were treated with SlowFade-Gold antifade reagent with DAPI (Invitrogen) and analysed by Fluorescence Microscopy with an Olympus IX51 Inverted Microscope.

Mouse assays

The ability of the recombinant YF viruses to elicit neutralizing antibodies directed against YF virus was evaluated by immunizing groups of 4 to 6 week old A/J mice subcutaneously with two doses of 100,000 PFU of either YF 17DD vaccine, YF17D/NS2B3/Tc, YF17D/ENS1/Tc or YF 17D Formulation 1 with an interval of 15 days. Two weeks after the last immunization, mice were bled and individual serum samples were treated 56ºC for 20 minutes. YF neutralizing antibody titer was determined by plaque reduction neutralization test (PRNT_50_). Titers were calculated as the highest dilution of antibody reducing 50% of the input virus plaques. Kruskall-Wallis test was used to compare the mean titers of neutralizing antibodies of the experimental groups.

To perform *T. cruzi* challenge assays, groups of five 4-6 week-old A/J mice were immunized with the different viruses as described above. Four weeks after the last dose, mice were challenged intraperitoneally with 250 bloodstream trypomastigotes of the *T. cruzi* Y strain. After challenge, clinical symptoms and deaths were daily recorded for a period of 60 days. Results represent pooled data from two independent experiments involving five to six animals per group and per experiment (except for YF17D/ENS1/Tc followed by YF17D/NS2B3/Tc, in the homologous prime-boost). Survival curves (Kaplan-Meyer method) were compared across groups and formally tested using the logrank test. Average survival times (AST) were compared between groups and tested by Mann Whitney test (GraphPad Prism 5.03 Program).

Parasitemia curves were determined by collecting blood from the tail vein daily after *T. cruzi* challenge and the level of parasitemia was calculated by Pizzi-Brener method . Results of parasitemia are representative of two independent experiments involving four to five animals per group. The values of peak parasitemia of each individual mouse were log transformed before being compared by one-way analysis of variance followed by Tukey´s test.

To establish whether the T.cruzi recombinant YF viruses could induce IFN-γ, groups of A/J mice were immunized as described above. One week after the first or the second dose, and two weeks after *T. cruzi* challenge, mice were sacrificed and the spleens removed. The IFN-γ ELISPOT assays were carried out using BD IFN-g ELISPOT set (BD – Biosciences Pharmingen, San Diego-CA). All ELISPOT assays were done as previously described . Spleen cells were incubated with medium alone, 10 μg/mL of synthetic TEWETGQI peptide (Invitrogen), 10,000 PFU/mL of inactivated YF 17DD virus or 4 μg of concanavalin A (Sigma) diluted in RPMI 10% FBS medium. Spots were counted with an Immunospot reader (Cellular Technology Ltd., Cleveland, OH) using the Immunospot Software version 3 by the ELISPOT Platform of PDTIS/Fiocruz - Rio de Janeiro . Statistical significance of the differences among immunization groups was verified by ANOVA Tukey´s test. Data are representative of two or more independent experiments.

**RESULTS**

**Design and characterization of recombinant 17D viruses expressing *T. cruzi* ASP-2_261-380_**

The ASP-2 _261-380_ fragment derived from Y strain of *T. cruzi* (Figure 1A) [35,37] was cloned into the intergenic E and NS1 region of the YF 17D genome [41] (Figure 1B), by means of the duplication of functional signalase cleavage motif as well as of conserved amino acid sequences flanking this region [41]. This structural arrangement allows the correct processing by cell signal peptidases in the ER membrane of the viral E and NS1 proteins as well as the recombinant ASP-2 protein with reduced disturbance in the virus replication cycle. The recombinant ASP-2_261-380_ cassette was predicted to be composed of 227 amino acids with an expected molecular weight of 24.7 kDa. The recombinant protein fragment ASP-2 contains two transmembrane helix domains corresponding to the transmembrane motifs present in the carboxi-terminus Den 4 E protein anchor region (TM1 and TM2). We could hypothetically corroborate the existence of these domains in the recombinant cassette by analysing the recombinant ASP-2 protein sequence with the TMHMM program for the prediction of transmembrane helices in proteins. Hence, the presence of these two transmembrane motifs in the recombinant cassete should promote the anchoring of the heterologous protein in the luminal side of the ER membrane, as we could demonstrate elsewhere . In this work, it was utilized a formerly described *T. cruzi* recombinant virus, denominated YF17D/NS2B3/Tc . This virus expresses the immunodominant ASP-2_320-327_ TEWETGQI epitope.

The recombinant YF/ ASP-2_261-380_ virus, identified as YF17D/ENS1/Tc was evaluated in terms of the replicative capability in comparison to the YF vaccine virus (substrain 17DD) and the other recombinant YF17D/NS2B3/Tc virus. The proliferation rate of the recombinant virus was lower than the vaccine counterpart (Figure 1C). The maximum virus yields occurred at 72 h post-infection with YF 17DD virus exhibiting a titer of 7.56 ± 0.07 log_10_PFU/mL while the recombinant YF17D/ENS1/Tc virus peaked at 96 h with a titer of 6.76 ± 0.35 log_10_PFU/mL. However, at these peak time points the differences between YF17D/ENS1/Tc and the control virus were not statistically significant (*P* value = 0.1). We observed a reduced plaque size of the recombinant virus YF 17D/ENS1/Tc as compared to the control vaccine YF 17DD virus (data not shown). We also compared the replication efficacy of the YF17D/ENS1/Tc but no statistically significant differences between them were seen despite that YF17D/NS2B3/Tc peaked earlier (at 72 h), similarly to YF 17DD virus, than the YF17D/ENS1/Tc virus (at 96 h) .

We also assessed the genetic stability of the YF17D/ENS1/Tc virus bearing the ASP-2_261-380_ gene, since the genetic stability is a concern for the development of vaccine viral vectors . In this regard, we carried out two series of separate serial passages in Vero cells per virus sample, infecting cells with a MOI of 0.02. At the second (2P) and sixth passage (6P), we performed RT-PCR and nucleotide sequencing analysis of amplicons containing the E / NS1 insertion region of the recombinant YF genome. In RT-PCR analysis, a DNA amplicon band of about 1,700 bp in size indicates the integrity of cassete region (Figure 1D, lanes 2 and 3). On the other hand, a DNA amplicon of 1,000 bp was obtained for the control virus YF 17DD, which contains no heterologous cassete (Figure 1D, lane 1). In conclusion, the integrity of the heterologous cassete was confirmed up to the sixth serial passage of YF17D/ENS1/Tc virus in Vero cells. Furthermore, we plaqued the recombinant virus belonging to the second and the sixth passages and no variation in plaque size was observed (data not shown).

**Detection of YF virus and *T. cruzi* antigens**

To confirm the expression of the heterologous *T. cruzi* ASP-2_261-380_ cassette by the recombinant YF17D/ENS1/Tc virus in infected Vero cells, we performed an indirect immunofluorescence assay using antibodies specific to TEWETGQI epitope (320 to 327 amino acids of ASP-2) or directed to YF antigens. The YF antigens were equally detected in Vero cells infected with vaccine 17D and the recombinant YF17D/ENS1/Tc and YF17D/NS2B3/Tc viruses (Figure 2A and B), whereas TEWETGQI epitope was specifically detected in the YF 17D/ENS1/Tc and YF17D/NS2B3/Tc virus infected cells (Figure 2D). Interestingly, the labeling pattern of YF 17D/ENS1/Tc in infected Vero cells was mainly localized in the perinuclear region suggesting that the heterologous recombinant protein is associated with the ER compartment, as previously observed for the Green Fluorescent Protein (GFP) using the same approach .

**Recombinant YF virus immunization induces antibodies to *T.cruzi* amastigotes**

Aiming to better characterize the YF17D/ENS1/Tc and YF17D/NS2B3/Tc viruses, we also performed immunofluorescence assays using sera from immunized A/J mice. We would like to establish if the vaccination with the recombinant viruses could elicit antibodies to the intracellular amastigote forms of *T. cruzi* in infected Vero cells. Remarkably, both viruses were able to induce antibodies against ASP-2 antigens, which could bind the ASP-2 on the amastigote cell surface in *T. cruzi* infected Vero cells (Figure 3A and B). A similar recognition profile could be obtained using control animal sera which received the TEWETGQI peptide emulsified with Freund´s Adjuvant (data not shown). Therefore, the immunization with the recombinant YF17D/ENS1/Tc and YF17D/NS2B3/Tc viruses could induce ASP-2 antibodies that are reactive to this protein in the context of the amastigote surface.

**Protective immune response against *T. cruzi***

To approach the use of these recombinant YF viruses as *T. cruzi* vaccine candidates, we immunizated A/J mice with them and challenged the animals with a lethal dose of *T. cruzi* trypomastigotes four weeks after from the last vaccine dose. The animal groups which were received either YF17D/NS2B3/Tc or YF17D/ENS1/Tc virus showed a reduction in mouse mortality of 16% and 25% at 30 dpi, respectively, while all the animals of the control group YF 17DD or Mock (data not shown) died (Figure 4A). Additionally, we could also observe an increment in the average survival time (AST) of these experimental groups: 26 days for mice vaccinated with YF17D/ENS1/Tc virus and of 27 days for those which received YF17D/NS2B3/Tc virus. In contrast, animals from the YF 17DD or Mock group displayed a significantly lower value of AST (20 days and 21 days, respectively; *P* < 0,0001, Mann Whitney test). However, these scores were not sustained after 60 dpi. Only 16% and 8% of A/J mice, which were immunized with recombinant ASP-2 _261-380_  or TEWETGQI virus, respectively, remained alive (Figure 4A).

Notwithstanding the low protection afforded by YF17D/ENS1/Tc or YF17D/NS2B3/Tc virus vaccination, we decided to test a mixture of these YF 17D recombinant viruses in mouse immunization. We wonder if a viral formulation could enhance the immunogenicity of viruses in A/J mice, perhaps promoting a more balanced MHC class I and II-restricted presentation and consequently to an improved immune-protective response. Using this rational, two different 17D viral compositions were utilized, Formulation 1 (composed of 50% of YF17D/NS2B3/Tc and 50% of YF17D/ENS1/Tc virus) and Formulation 2 (composed of 75% of YF17D/ENS1/Tc and 25% of YF17D/NS2B3/Tc). Notably, groups of animal vaccinated with YF 17D viral formulations exhibited a higher percentage of survivors after the lethal *T. cruzi* challenge. Formulation 1 protected 58% of mice at 60 dpi, with an AST of 29 days (Figure 4A) while Formulation 2 protected 43% of mice at 60 dpi, with an AST of 24 days (data not shown). This enhancement of the AST was statistically significant in comparison for the group vaccinated with YF 17DD (*P* < 0.01).

To determine if a heterologous prime-boost vaccination sequentially using the recombinant viruses could lead to the same degree of protection provided by the formulations, we first vaccinated mice with the YF17D/ENS1/Tc virus (first dose) and subsequently, with the YF17D/NS2B3/Tc virus (second dose). We verified that only 9% of A/J mice vaccinated with the prime-boost regimen was protected against the lethal *T. cruzi* challenge at 60 dpi (data not shown). Therefore, the sequencial immunization strategy (heterologous prime-boost) was not able to elicit the same level of protection as the co-administration of both recombinant viruses (viral formulation).

We also analysed the parasitemia in challenged animals that were previously immunized with Formulation 1, since it could display the most favourable degree of protection regarding the Formulation 2 and the homologous prime-boost regimen. So, we chose it to study the *T. cruzi* parasitemia comparing to the two vaccines composed by only a single recombinant virus. Corroborating the effect on the decrease of mortality and the increase of AST, mice vaccinated with Formulation 1 presented a lower parasitemia (average of 120 x 10^4^ parasites/mL) than the animals vaccinated with YF 17DD (410 x 10^4^ parasites/mL) or YF17D/NS2B3/Tc (360 x 10^4^ parasites/mL) (*P* < 0.05) (Figure 4B). However, no significant difference was observed when we compared the peak parasitemias of mice immunized with YF17D/ENS1/Tc (185 x 10^4^ parasites/mL) or Formulation 1 (120 x 10^4^ parasites/mL). The individual mouse values per immunizing group are shown in Figure 4C to F.

**Production of neutralizing antibodies to Yellow Fever Virus**

One important point to be considered in the efficacy of recombinant YF vaccines is the ability to elicit neutralizing antibodies to YF virus. So, we decided to compare the ability of these different immunization regimens to induce this kind of immune response against YF virus. Remarkably, when we compared the distinct recombinant YF groups to the control vaccine 17DD virus, there was a general decrease in titers of neutralizing antibodies found in mouse groups vaccinated with any recombinant virus vaccine. Thus, in YF 17D/ENS1/Tc virus vaccinated group, despite the seroconversion rate of 100%, the titers of neutralizing antibodies to YF were significantly lower when compared to the YF 17DD group, displaying titers ranging from 1:18 to 1:235; with an average titer (GMT) of 1:69, while the vaccine control group ranged from 1:99 to more than 1:1,280; with a GMT of 1:559 (Table 1). This difference in YF neutralizing antibody production was statistically significant (*P* < 0.0001) suggesting that the YF 17D/ENS1/Tc virus might be more attenuated *in vivo*.

Despite the fact that the virus formulation seemed to be more immunogenic in challenge experiments, it caused a partial seroconversion ratio of immunized A/J mice and was not able to induce similar antibody titers as the vaccine YF 17D virus was (Table 1). The titers obtained for Formulation 1 ranged from 1:18 to 1:86 and displayed a GMT of 1:51 while for Formulation 2 it varied from 1:10 to 1:82 with a GMT of 1:37. There was no significant difference between them. In contrast, antibody titers induced by TEWETGQI expressing virus, YF 17D/NS2B3/Tc, were significantly higher than both viral formulations (*P* < 0.05) and did not exhibit difference to the titer found in the YF 17DD control group.

**Vaccination with YF virus formulation induces IFN-γ mediated-cellular immune responses in A/J mice**

To better characterize the protection promoted by YF 17D Formulation 1 immunization, we collected the spleens from the animals one week after the first and the second dose of vaccination and determined the amount of IFN-γ-secreting splenocytes upon stimulation with *T. cruzi* T CD8^+^ TEWETGQI epitope or inactivated YF 17DD virus.

Seven days after the first vaccine dose, we observed that all immunization groups, i.e., single recombinant virus vaccines, Formulation 1 and YF 17DD vaccine control were not able to induce a considerable response of IFN-γ spot forming-cells (SFC) specific to yellow fever since there were no statistically differences among the number of IFN-γ secreting splenocytes between vaccinated groups and the mock group (Figure 5A). However, after the second dose, the numbers of IFN-γ-secreting splenocytes significantly increased for all immunized mouse groups compared to the negative control group (*P* < 0.05). Noteworthy, the YF vaccinated groups did not exhibit any difference in the amount of IFN-γ-secreting splenocytes after the second dose (159 SFC /10^6^ cells for YF 17DD, 149 SFC /10^6^ cells for YF 17D/NS2B3/Tc, 215 SFC / 10^6^ cells for YF 17D/ENS1/Tc and 151 SFC /10^6^ cells for Formulation 1; Figure 5B). Therefore, the cellular responses observed for all groups indicated an equivalent capacity for eliciting and expanding specific T cells against the YF virus antigens (Figure 5B).

In the next study, we investigated the ability of the recombinant *T.cruzi* YF viruses to elicit IFN-γ-producing cells specific to the immunodominant H-2K^k^ - TEWETGQI epitope of ASP-2 in A/J mice before and after *T. cruzi* trypomastigote challenge. Interestingly, after a single dose, we detected a significantly higher number of SFCs in mice immunized with Formulation 1 (95 SFC / 10^6^ cells) in comparison to YF 17DD (1 SFC / 10^6^ cells), Mock (4 SFC / 10^6^ cells) or YF 17D/ENS1/Tc (37 SFC / 10^6^ cells) (*P* < 0.05; Figure 5C). The YF17D/NS2B3/Tc virus was also capable to elicit statistically significant T cell response to the *T. cruzi* antigen after one dose (41 SFC / 10^6^ cells) in comparison to YF 17DD or Mock (*P* < 0.05) although not in the same magnitude as Formulation 1 (95 SFC / 10^6^ cells). After the second dose, we could observe an overall expansion of the specific TEWETGQI-peptide IFN-γ-producing cells in mice immunized with YF 17D/NS2B3/Tc, YF 17D/ENS1/Tc, and Formulation 1, reaching 135, 234 and 223 SFC / 10^6^ cells, respectively, as compared to the YF 17DD group which exhibited 6 SFC / 10^6^ cells (Figure 5D). All in all, it is reasonable to consider that the YF 17DD virus as well as the recombinant *T. cruzi* YF virus, including the Formulation 1, displayed a similar ability to induce a IFN-γ immune cell response specific to YF virus after two doses of vaccination, whereas this kind of immune response specific to the TEWETGQI peptide was higher for Formulation 1 and YF 17D/ENS1/Tc immunized-groups in comparison to the YF 17DD, Mock and the YF 17D/NS2B3/Tc groups. These data show a more divergent aptitude in eliciting cellular responses specifically directed to the TEWETGQI epitope than to the Yellow Fever Virus specific epitopes (although YF 17D/ENS1/Tc could induce higher TEWETGQI or YF specific cellular responses than YF17D/NS2B3/Tc virus after two doses).

After *T. cruzi* challenge, higher numbers of IFN-γ SFC were achieved in the groups immunized with Formulation 1 (1661 SFC / 10^6^ cells) followed by the YF 17D/ENS1/Tc (1634 SFC / 10^6^ cells) and the YF 17D/NS2B3/Tc virus (1030 SFC / 10^6^ cells) in comparison to the YF 17DD group (94 SFC / 10^6^ cells) or Mock (1 SFC/ 10^6^ cells, *P* < 0.05; Figure 5E). Therefore, the number of IFN-γ producing T cells specific to the TEWETGQI epitope obtained from the splenocytes of mice immunized with each of the YF recombinant viruses or in combination (Formulation 1) was up to six-fold higher after encountering the parasite antigens suggesting that the recombinant viruses were capable to elicit and considerably expand specific T cells after a *T. cruzi* challenge (Figure 5E).

**DISCUSSION**

Despite considerable efforts over the last decades, the development of a *Trypanosoma cruzi* vaccine remains elusive. The use of viral vectors may be one of the promising strategies to elicit protective immune responses against this parasite, which may require the activation of a Th1 immune profile, with the stimulation of CD8^+^ T cells. Given the outstanding properties of the YF 17D virus with regard to the CD8^+^ T cell response we consider that YF 17D virus is an appropriate vector to deliver *T. cruzi* epitopes and generate the required immune response for an ideal anti-parasitic vaccine.

Amastigote surface protein 2 is expected to be surface anchored by glycosylphosphatidylinositol (GPI) structures and may be released into the host cell cytoplasm throughout the cycle of intracellular development of the parasite . ASP-2 harbors many major histocompatibility complex (MHC) class I-restricted epitopes shown to be recognized by specific cytotoxic T cells derived from mice or man . These specific CD8^+^ T-cells directed to amastigote forms can lyse *T. cruzi* infected cells and produce important pro-inflammatory cytokines such as IFN-γ that may help to eliminate intracellular parasites . In addition, immunization with fragments or peptides derived from ASP-2 has been demonstrated to generate specific type 1 IFN-γ producing and cytotoxic T cells in vaccinated mice. For these reasons, ASP-2 is considered one of the best targets for an effective host response.

We have previously demonstrated that vaccination of a high susceptible mice model, A/J, with recombinant YF 17D viruses expressing an ASP-2 CD8^+^ T cell epitope, TEWETGQI, induced specific IFN-γ producing-cells but only partial protection of mice after a *T. cruzi* challenge . In this study, we have used the previously described YF virus platform of insertion between the E protein and the non-structural NS1 protein to express a larger immunogenic fragment of *T. cruzi* ASP-2 protein including the TEWETGQI epitope, resulting in the YF 17D/ENS1/Tc virus ,. This viral construct is stable since the integrity of the expression cassette was maintained until at least the sixth serial passage in Vero cells. However, this virus exhibited reduced fitness *in vivo* since it elicited lower titers of neutralizing antibodies as compared to animals vaccinated with YF 17DD virus. We are aware that studies to determine antigenic mass production in viremic animals are necessary to correlate the over-attenuation of YF 17D/ENS1/Tc with its lower immunogenicity as measured by neutralizing antibodies to YF. Nevertheless, it has been shown that even low detectable levels of YF 17D viremia (less than 18 copies/mL) did not impair the induction of T cell immune response (up to 388 SFU/10^6^ cells) in vaccinated Indian rhesus macaques .

In our studies in A/J mice, two doses of YF 17D/ENS1/Tc virus elicited antibodies against amastigote forms of *T. cruzi* and induced a significant number of IFN-γ producing-T cells directed to the *T. cruzi* TEWETGQI peptide. However, there was no complete protection against a lethal *T. cruzi* challenge which contrasts to other studies that described 100% of protection when mice were vaccinated with the recombinant protein expressing the same antigens derived from ASP-2 in the presence of CpG ODN 1826 adjuvant. Although the regimens of immunization greatly contrast, it is possible that administration of this adjuvant, a Toll-like receptor 9 agonist, is enhancing protective CD8^+^ T cell response in those mice . We might also consider that the major retention of ASP-2_261-380_ antigens in the ER, as expressed by the YF17D/ENS1/Tc virus may contribute to a restricted presentation of antigens derived from this ASP-2 fragment via MHC class I.

A recent report has shown that the utilization of distinct viral vectors as a single mixture composition rather than as heterologous prime-boost regimen is a more promising and effective vaccine strategy allowing enhanced protection against pre-erythrocytic malaria in a mouse model . Given the low immunogenicity of the YF17D/ENS1/Tc virus, perhaps due to the fact that the expressed the heterologous ASP-2 antigen by YF 17D/ENS1/Tc virus is retained into the ER compartment, we introduced a new immunization strategy to achieve a better immune response and higher level of protection of A/J mice against *T. cruzi* challenge. In this regard, we used YF 17D viral formulations that consisted of two YF 17D recombinant viruses (YF17D/ENS1/Tc and YF17D/NS2B3/Tc), to vaccinate A/J mice. The choice of using YF17D/NS2B3/Tc recombinant virus simultaneously with YF17D/ENS1/Tc was based on the fact that the former virus was highly immunogenic, capable to produce high titers of neutralizing antibodies to YF virus and a strong IFN-γ cellular response to Yellow Fever Virus and the *T. cruzi* TEWETGQI epitope in vaccinated mice. Also, the two viruses carry in their genomic sequences the CD8^+^ T cell epitope TEWETGQI, previously described as a protective immunogen ,.

Our results indicated that Formulation 1 (composed of 50% of each virus) was capable to protect nearly 60% of challenged A/J mice by day 60 post-challenge with reduced parasitemia levels after trypomastigote challenge in comparison to the immunization with YF17D/ENS1/Tc or YF17D/NS2B3/Tc recombinant viruses alone. It has been proposed that a more effective CD8^+^ T cell recall responses to control re-invading pathogens depend on the amplitude of the previous CD8^+^ T cell burst in the primary infection, because it can generate a higher number of memory T cells . We demonstrated that Formulation 1 induced a significantly higher number of IFN-γ producing-T cells specific to TEWETGQI after only one dose in A/J mice. Although the immune correlates of protection are still undefined, these findings suggest that an early T cell activation might correlate with an improved protective immune response. Further analysis of TEWETGQI-specific effector and memory CD8^+^ T cell phenotypes elicited by Formulation 1 after the first dose should be carried out in order to confirm this correlation.

We also noted that the recombinant viruses and Formulation 1 were similarly capable to improve the number of IFN-γ producing-T cells specific to YF peptides or TEWETGQI epitope after a second dose and induce a significant anamnestic response to TEWETGQI after a *T. cruzi* challenge. In this sense, our results suggest that the early T cell response detected after a single dose of Formulation 1 might better correlate with the immune protection levels observed whereas T cell expansion of all viruses groups detected 15 days after challenge seem to be associated with the initial resistance of mice to succumb after a lethal *T. cruzi* infection instead of a long lasting protection. Notwithstanding the fact that both YF17D/ENS1/Tc virus and Formulation 1 could control the parasitemia in the acute phase of *T. cruzi* infection, only Formulation 1 provided a superior, although partial, protection of mice.

Some of the possible explanations for the improved level of immunogenicity induced by Formulation 1 may be that the TEWETGQI epitope, being presented by both viruses through different cell compartments: cytoplasm (released by NS2B/NS3 protease cleavage processing) or ER lumen (mediated by anchoring of the C-terminus of Den 4 E protein domains) is able to induce distinct pathways of presentation *in vivo*, enhancing the immune response. Another relevant matter is that YF17D/ENS1/Tc harbors parasite-derived epitopes other than TEWETGQI. As previously suggested , it is possible that the presence of CD4^+^ T cell epitopes in ASP-2 fragments may impact on the priming of protective CD8^+^ T cells. In this sense, we speculate that other epitopes as yet unidentified in the ASP-2_261-380_ fragment expressed by the YF17D/ENS1/Tc virus may also have helped the induction of protective TEWETGQI-specific CD8^+^ T cells.

It is well-known that accumulation of incorrectly folded proteins inside the ER is a source of cellular stress, leading to apoptosis . A plausible explanation of how the YF17D/ENS1/Tc virus presents ASP-2 epitopes via MHC class II to CD4^+^ T target cells is that dendritic cells (DC) engulf the apoptotic bodies that are produced in cellular stress and may present antigens to CD4^+^ T cells . The cross-presentation of ASP-2 epitopes via MHC class I to CD8^+^ T cells by APCs that have phagocited apoptotic bodies or by ER-associated degradation mechanism might also contribute to improve the CD8 T cell response. In face of these possible mechanisms, we propose that the CD8^+^ TEWETGQI epitope presentation by the YF17D/ENS1/Tc virus is being complemented by the direct cytosol MHC class I-restricted presentation of TEWETGQI by the YF17D/NS2B3/Tc virus in YF 17D Formulation 1 immunizations, resulting in a stronger T CD8^+^ response.

Nevertheless, we verified that YF 17D viral formulation 1 did not induce high titers of neutralizing antibodies against YF. However, Formulation 1 immunization was capable to induce T cell immune response directed to YF peptides, as observed in ELISPOT assays, in similar levels to the animal groups that received the YF 17DD virus vaccine. These results demonstrated that the production of neutralizing antibodies to the YF virus may not always correlate with the generation of CD8^+^ T cell response. This observation may be due to the fact that antibody production is directly dependent on the binding of the antigen to the B cell receptor, being therefore, also dependent on virus spreading *in vivo* .

This preliminary result is in accordance with previous studies in which YF17D recombinant viruses were shown to elicit a potent T-cell response . However, the polyfunctionality of these YF 17D recombinant virus induced cells should be studied by analysing their cytokine synthesis profiles as well as the functional capabilities of effector and memory T cell responses, since these parameters can be associated to mechanisms of defense against pathogens .

We have studied both arms of the immune response that is, humoral (neutralizing antibody levels) and cellular (production of IFN gamma) and both revealed aspects characteristic of YF 17D vaccine and YF 17D recombinant viruses. We anticipate extending such evaluation by performing a series of phenotypic analyses of T CD8^+^ lymphocyte markers such as T-cell degranulation and cytotoxicity CD107a marker, KLRG-1 activation marker as well as surface markers that differentiate T effector-memory and T central memory subpopulations such as CD44, CD62L and CCR7 on the cellular response and its importance for protection against challenge. It will also be important to study the CD4^+^ Th1 cells since they are particularly important to the development of acquired immunity against experimental infection in mice with protozoan parasites. In addition we intend to study the immunoglobulin class in the humoral response as the IgG class is important for viral clearance. The role of antibodies in *T. cruzi* infections is not clear but for the immune response to the vector is of utmost relevance and may be useful to validate the 17D virus platform.

In this regard, our findings demonstrate that the co-administration of recombinant yellow fever viruses that differentially express heterologous antigens of a same protein can elicit a superior immune response in a mouse model. Inclusion of other immunogenic *T. cruzi* ASP-2 antigens in new optimized platforms of YF 17D virus and the use of innovative YF 17D viral formulations may broaden the immunogenicity of YF 17D recombinant viruses. This should expand the applicability of YF 17D as a potential recombinant virus vector to express other antigens for Chagas’ disease vaccine development as well as antigens from other pathogens causing diseases of interest.

**Acknowledgements**

We would like to express our gratitude for Fernanda Rimolli, Luiz da Silva and Isabel Freire from Laboratório de Experimentação Animal – Bio-Manguinhos for technical assistance on mouse experimental studies, Gisela F. Trindade for fluorescence microscopic assays and Heloisa Diniz for image editing. The authors are also in debt to PDTIS-FIOCRUZ for supporting the sequencing, ELISpot and confocal microscopy studies through the Genomics and Confocal Microscopy Facilities. This work was supported by grants from CNPq, PDTIS/FIOCRUZ (RVR03), Instituto Nacional de Ciência e Tecnologia em Vacinas (INCTV- CNPq/MCT). MCB, RG and RTN were recipients of fellowships from CNPq.

**REFERENCES**

1. Rassi A, Marcondes de Rezende J (2012) American trypanosomiasis (Chagas disease). Infect Dis Clin North Am 26: 275-291.

2. Schmunis GA, Yadon ZE (2010) Chagas disease: a Latin American health problem becoming a world health problem. Acta Trop 115: 14-21.

3. Albajar-Vinas P, Jannin J (2011) The hidden Chagas disease burden in Europe. Euro Surveill 16.

4. Quijano-Hernandez I, Dumonteil E (2011) Advances and challenges towards a vaccine against Chagas disease. Hum Vaccin 7: 1184-1191.

5. Gulland A (2012) Plan to stimulate research in developing countries is put on hold. BMJ 344: e3771.

6. Rodrigues M, de Alencar B, Claser C, Tzelepis F, Silveira E, et al. (2009) Swimming against the current: genetic vaccination against Trypanosoma cruzi infection in mice. Mem Inst Oswaldo Cruz 104 Suppl 1: 281-287.

7. Dumonteil E (2009) Vaccine development against Trypanosoma cruzi and Leishmania species in the post-genomic era. Infect Genet Evol 9: 1075-1082.

8. Cazorla SI, Frank FM, Malchiodi EL (2009) Vaccination approaches against Trypanosoma cruzi infection. Expert Rev Vaccines 8: 921-935.

9. Junqueira C, Caetano B, Bartholomeu DC, Melo MB, Ropert C, et al. (2010) The endless race between Trypanosoma cruzi and host immunity: lessons for and beyond Chagas disease. Expert Rev Mol Med 12: e29.

10. Lee BY, Bacon KM, Connor DL, Willig AM, Bailey RR (2010) The potential economic value of a Trypanosoma cruzi (Chagas disease) vaccine in Latin America. PLoS Negl Trop Dis 4: e916.

11. Martin D, Tarleton R (2004) Generation, specificity, and function of CD8+ T cells in Trypanosoma cruzi infection. Immunol Rev 201: 304-317.

12. Rodrigues M, Boscardin S, Vasconcelos J, Hiyane M, Salay G, et al. (2003) Importance of CD8 T cell-mediated immune response during intracellular parasitic infections and its implications for the development of effective vaccines. An Acad Bras Cienc 75: 443-468.

13. Martin DL, Tarleton RL (2005) Antigen-specific T cells maintain an effector memory phenotype during persistent Trypanosoma cruzi infection. J Immunol 174: 1594-1601.

14. Hoft D, Eickhoff C (2005) Type 1 immunity provides both optimal mucosal and systemic protection against a mucosally invasive, intracellular pathogen. Infect Immun 73: 4934-4940.

15. Dominguez MR, Silveira EL, de Vasconcelos JR, de Alencar BC, Machado AV, et al. (2011) Subdominant/cryptic CD8 T cell epitopes contribute to resistance against experimental infection with a human protozoan parasite. PLoS One 6: e22011.

16. Laucella S, Postan M, Martin D, Hubby Fralish B, Albareda M, et al. (2004) Frequency of interferon- gamma -producing T cells specific for Trypanosoma cruzi inversely correlates with disease severity in chronic human Chagas disease. J Infect Dis 189: 909-918.

17. Aliberti JC, Souto JT, Marino AP, Lannes-Vieira J, Teixeira MM, et al. (2001) Modulation of chemokine production and inflammatory responses in interferon-gamma- and tumor necrosis factor-R1-deficient mice during Trypanosoma cruzi infection. Am J Pathol 158: 1433-1440.

18. Silva JS, Vespa GN, Cardoso MA, Aliberti JC, Cunha FQ (1995) Tumor necrosis factor alpha mediates resistance to Trypanosoma cruzi infection in mice by inducing nitric oxide production in infected gamma interferon-activated macrophages. Infect Immun 63: 4862-4867.

19. de Alencar B, Persechini P, Haolla F, de Oliveira G, Silverio J, et al. (2009) Perforin and gamma interferon expression are required for CD4+ and CD8+ T-cell-dependent protective immunity against a human parasite, Trypanosoma cruzi, elicited by heterologous plasmid DNA prime-recombinant adenovirus 5 boost vaccination. Infect Immun 77: 4383-4395.

20. Gutierrez FR, Mineo TW, Pavanelli WR, Guedes PM, Silva JS (2009) The effects of nitric oxide on the immune system during Trypanosoma cruzi infection. Mem Inst Oswaldo Cruz 104 Suppl 1: 236-245.

21. Tzelepis F, de Alencar B, Penido M, Gazzinelli R, Persechini P, et al. (2006) Distinct kinetics of effector CD8+ cytotoxic T cells after infection with Trypanosoma cruzi in naive or vaccinated mice. Infect Immun 74: 2477-2481.

22. Pérez Brandan C, Basombrío MA (2012) Genetically attenuated Trypanosoma cruzi parasites as a potential vaccination tool. Bioengineered 3.

23. Cazorla S, Frank F, Becker P, Arnaiz M, Mirkin G, et al. (2010) Redirection of the immune response to the functional catalytic domain of the cystein proteinase cruzipain improves protective immunity against Trypanosoma cruzi infection. J Infect Dis 202: 136-144.

24. Eickhoff CS, Giddings OK, Yoshida N, Hoft DF (2010) Immune responses to gp82 provide protection against mucosal Trypanosoma cruzi infection. Mem Inst Oswaldo Cruz 105: 687-691.

25. Araújo A, de Alencar B, Vasconcelos J, Hiyane M, Marinho C, et al. (2005) CD8+-T-cell-dependent control of Trypanosoma cruzi infection in a highly susceptible mouse strain after immunization with recombinant proteins based on amastigote surface protein 2. Infect Immun 73: 6017-6025.

26. Hoft DF, Eickhoff CS, Giddings OK, Vasconcelos JR, Rodrigues MM (2007) Trans-sialidase recombinant protein mixed with CpG motif-containing oligodeoxynucleotide induces protective mucosal and systemic trypanosoma cruzi immunity involving CD8+ CTL and B cell-mediated cross-priming. J Immunol 179: 6889-6900.

27. Duan X, Yonemitsu Y, Chou B, Yoshida K, Tanaka S, et al. (2009) Efficient protective immunity against Trypanosoma cruzi infection after nasal vaccination with recombinant Sendai virus vector expressing amastigote surface protein-2. Vaccine 27: 6154-6159.

28. Machado AV, Cardoso JE, Claser C, Rodrigues MM, Gazzinelli RT, et al. (2006) Long-term protective immunity induced against Trypanosoma cruzi infection after vaccination with recombinant adenoviruses encoding amastigote surface protein-2 and trans-sialidase. Hum Gene Ther 17: 898-908.

29. Miyahira Y, Takashima Y, Kobayashi S, Matsumoto Y, Takeuchi T, et al. (2005) Immune responses against a single CD8+-T-cell epitope induced by virus vector vaccination can successfully control Trypanosoma cruzi infection. Infect Immun 73: 7356-7365.

30. Takayama E, Ono T, Carnero E, Umemoto S, Yamaguchi Y, et al. (2010) Quantitative and qualitative features of heterologous virus-vector-induced antigen-specific CD8+ T cells against Trypanosoma cruzi infection. Int J Parasitol 40: 1549-1561.

31. Rigato PO, de Alencar BC, de Vasconcelos JR, Dominguez MR, Araújo AF, et al. (2011) Heterologous plasmid DNA prime-recombinant human adenovirus 5 boost vaccination generates a stable pool of protective long-lived CD8(+) T effector memory cells specific for a human parasite, Trypanosoma cruzi. Infect Immun 79: 2120-2130.

32. Limon-Flores AY, Cervera-Cetina R, Tzec-Arjona JL, Ek-Macias L, Sánchez-Burgos G, et al. (2010) Effect of a combination DNA vaccine for the prevention and therapy of Trypanosoma cruzi infection in mice: role of CD4+ and CD8+ T cells. Vaccine 28: 7414-7419.

33. Sanchez-Burgos G, Mezquita-Vega RG, Escobedo-Ortegon J, Ramirez-Sierra MJ, Arjona-Torres A, et al. (2007) Comparative evaluation of therapeutic DNA vaccines against Trypanosoma cruzi in mice. FEMS Immunol Med Microbiol 50: 333-341.

34. Cazorla S, Frank F, Becker P, Corral R, Guzmán C, et al. (2008) Prime-boost immunization with cruzipain co-administered with MALP-2 triggers a protective immune response able to decrease parasite burden and tissue injury in an experimental Trypanosoma cruzi infection model. Vaccine 26: 1999-2009.

35. Morell M, Thomas MC, Caballero T, Alonso C, López MC (2006) The genetic immunization with paraflagellar rod protein-2 fused to the HSP70 confers protection against late Trypanosoma cruzi infection. Vaccine 24: 7046-7055.

36. Egui A, Thomas MC, Morell M, Marañón C, Carrilero B, et al. (2012) Trypanosoma cruzi paraflagellar rod proteins 2 and 3 contain immunodominant CD8(+) T-cell epitopes that are recognized by cytotoxic T cells from Chagas disease patients. Mol Immunol 52: 289-298.

37. Taibi A, Plumas-Marty B, Guevara-Espinoza A, Schöneck R, Pessoa H, et al. (1993) Trypanosoma cruzi: immunity-induced in mice and rats by trypomastigote excretory-secretory antigens and identification of a peptide sequence containing a T cell epitope with protective activity. J Immunol 151: 2676-2689.

38. Planelles L, Thomas MC, Alonso C, López MC (2001) DNA immunization with Trypanosoma cruzi HSP70 fused to the KMP11 protein elicits a cytotoxic and humoral immune response against the antigen and leads to protection. Infect Immun 69: 6558-6563.

39. Vasconcelos J, Hiyane M, Marinho C, Claser C, Machado A, et al. (2004) Protective immunity against trypanosoma cruzi infection in a highly susceptible mouse strain after vaccination with genes encoding the amastigote surface protein-2 and trans-sialidase. Hum Gene Ther 15: 878-886.

40. Garg N, Tarleton R (2002) Genetic immunization elicits antigen-specific protective immune responses and decreases disease severity in Trypanosoma cruzi infection. Infect Immun 70: 5547-5555.

41. de Alencar B, Araújo A, Penido M, Gazzinelli R, Rodrigues M (2007) Cross-priming of long lived protective CD8+ T cells against Trypanosoma cruzi infection: importance of a TLR9 agonist and CD4+ T cells. Vaccine 25: 6018-6027.

42. Dutra WO, Menezes CA, Villani FN, da Costa GC, da Silveira AB, et al. (2009) Cellular and genetic mechanisms involved in the generation of protective and pathogenic immune responses in human Chagas disease. Mem Inst Oswaldo Cruz 104 Suppl 1: 208-218.

43. Silveira E, Claser C, Haolla F, Zanella L, Rodrigues M (2008) Novel protective antigens expressed by Trypanosoma cruzi amastigotes provide immunity to mice highly susceptible to Chagas' disease. Clin Vaccine Immunol 15: 1292-1300.

44. Martin D, Weatherly D, Laucella S, Cabinian M, Crim M, et al. (2006) CD8+ T-Cell responses to Trypanosoma cruzi are highly focused on strain-variant trans-sialidase epitopes. PLoS Pathog 2: e77.

45. Theiler M, Smith HH (2000) The use of yellow fever virus modified by in vitro cultivation for human immunization. J. Exp. Med. 65, 787-800 (1937). Rev Med Virol 10: 6-16; discussion 13-15.

46. Poland J, Calisher C, Monath T, Downs W, Murphy K (1981) Persistence of neutralizing antibody 30-35 years after immunization with 17D yellow fever vaccine. Bull World Health Organ 59: 895-900.

47. Lefeuvre A, Marianneau P, Deubel V (2004) Current Assessment of Yellow Fever and Yellow Fever Vaccine. Curr Infect Dis Rep 6: 96-104.

48. Querec T, Bennouna S, Alkan S, Laouar Y, Gorden K, et al. (2006) Yellow fever vaccine YF-17D activates multiple dendritic cell subsets via TLR2, 7, 8, and 9 to stimulate polyvalent immunity. J Exp Med 203: 413-424.

49. Stoyanov C, Boscardin S, Deroubaix S, Barba-Spaeth G, Franco D, et al. (2010) Immunogenicity and protective efficacy of a recombinant yellow fever vaccine against the murine malarial parasite Plasmodium yoelii. Vaccine 28: 4644-4652.

50. Franco D, Li W, Qing F, Stoyanov C, Moran T, et al. (2010) Evaluation of yellow fever virus 17D strain as a new vector for HIV-1 vaccine development. Vaccine 28: 5676-5685.

51. Co M, Terajima M, Cruz J, Ennis F, Rothman A (2002) Human cytotoxic T lymphocyte responses to live attenuated 17D yellow fever vaccine: identification of HLA-B35-restricted CTL epitopes on nonstructural proteins NS1, NS2b, NS3, and the structural protein E. Virology 293: 151-163.

52. van der Most RG, Harrington LE, Giuggio V, Mahar PL, Ahmed R (2002) Yellow fever virus 17D envelope and NS3 proteins are major targets of the antiviral T cell response in mice. Virology 296: 117-124.

53. Miller J, van der Most R, Akondy R, Glidewell J, Albott S, et al. (2008) Human effector and memory CD8+ T cell responses to smallpox and yellow fever vaccines. Immunity 28: 710-722.

54. Monath T, Barrett A (2003) Pathogenesis and pathophysiology of yellow fever. Adv Virus Res 60: 343-395.

55. Neves PC, Rudersdorf RA, Galler R, Bonaldo MC, de Santana MG, et al. (2010) CD8+ gamma-delta TCR+ and CD4+ T cells produce IFN-γ at 5-7 days after yellow fever vaccination in Indian rhesus macaques, before the induction of classical antigen-specific T cell responses. Vaccine 28: 8183-8188.

56. Tzelepis F, Persechini P, Rodrigues M (2007) Modulation of CD4(+) T cell-dependent specific cytotoxic CD8(+) T cells differentiation and proliferation by the timing of increase in the pathogen load. PLoS One 2: e393.

57. Nogueira RT, Nogueira AR, Pereira MC, Rodrigues MM, Galler R, et al. (2011) Biological and immunological characterization of recombinant Yellow Fever 17D viruses expressing a Trypanosoma cruzi Amastigote Surface Protein-2 CD8+ T cell epitope at two distinct regions of the genome. Virol J 8: 127.

58. Bonaldo M, Mello S, Trindade G, Rangel A, Duarte A, et al. (2007) Construction and characterization of recombinant flaviviruses bearing insertions between E and NS1 genes. Virol J 4: 115.

59. Bonaldo M, Martins M, Rudersdorf R, Mudd P, Sacha J, et al. (2010) Recombinant yellow fever vaccine virus 17D expressing simian immunodeficiency virus SIVmac239 gag induces SIV-specific CD8+ T-cell responses in rhesus macaques. J Virol 84: 3699-3706.

60. Boscardin S, Kinoshita S, Fujimura A, Rodrigues M (2003) Immunization with cDNA expressed by amastigotes of Trypanosoma cruzi elicits protective immune response against experimental infection. Infect Immun 71: 2744-2757.

61. Bonaldo M, Garratt R, Caufour P, Freire M, Rodrigues M, et al. (2002) Surface expression of an immunodominant malaria protein B cell epitope by yellow fever virus. J Mol Biol 315: 873-885.

62. Rice C, Grakoui A, Galler R, Chambers T (1989) Transcription of infectious yellow fever RNA from full-length cDNA templates produced by in vitro ligation. New Biol 1: 285-296.

63. Brener Z (1969) The behavior of slender and stout forms of Trypanosoma cruzi in the blood-stream of normal and immune mice. Ann Trop Med Parasitol 63: 215-220.

64. Lima-Junior JC, Tran TM, Meyer EV, Singh B, De-Simone SG, et al. (2008) Naturally acquired humoral and cellular immune responses to Plasmodium vivax merozoite surface protein 9 in Northwestern Amazon individuals. Vaccine 26: 6645-6654.

65. Liniger M, Zuniga A, Naim HY (2007) Use of viral vectors for the development of vaccines. Expert Rev Vaccines 6: 255-266.

66. Low HP, Tarleton RL (1997) Molecular cloning of the gene encoding the 83 kDa amastigote surface protein and its identification as a member of the Trypanosoma cruzi sialidase superfamily. Molecular and biochemical parasitology 88: 137-149.

67. Low HP, Santos MA, Wizel B, Tarleton RL (1998) Amastigote surface proteins of Trypanosoma cruzi are targets for CD8+ CTL. Journal of immunology 160: 1817-1823.

68. Wizel B, Palmieri M, Mendoza C, Arana B, Sidney J, et al. (1998) Human infection with Trypanosoma cruzi induces parasite antigen-specific cytotoxic T lymphocyte responses. The Journal of clinical investigation 102: 1062-1071.

69. Rodrigues MM, Boscardin SB, Vasconcelos JR, Hiyane MI, Salay G, et al. (2003) Importance of CD8 T cell-mediated immune response during intracellular parasitic infections and its implications for the development of effective vaccines. Anais da Academia Brasileira de Ciencias 75: 443-468.

70. Mudd P, Piaskowski S, Neves P, Rudersdorf R, Kolar H, et al. (2010) The live-attenuated yellow fever vaccine 17D induces broad and potent T cell responses against several viral proteins in Indian rhesus macaques--implications for recombinant vaccine design. Immunogenetics 62: 593-600.

71. Reyes-Sandoval A, Rollier CS, Milicic A, Bauza K, Cottingham MG, et al. (2012) Mixed Vector Immunization With Recombinant Adenovirus and MVA Can Improve Vaccine Efficacy While Decreasing Antivector Immunity. Mol Ther.

72. Miyahira Y (2008) Trypanosoma cruzi infection from the view of CD8+ T cell immunity--an infection model for developing T cell vaccine. Parasitol Int 57: 38-48.

73. Todd DJ, Lee AH, Glimcher LH (2008) The endoplasmic reticulum stress response in immunity and autoimmunity. Nat Rev Immunol 8: 663-674.

74. Green DR, Ferguson T, Zitvogel L, Kroemer G (2009) Immunogenic and tolerogenic cell death. Nat Rev Immunol 9: 353-363.

75. Heath W, Carbone F (2001) Cross-presentation in viral immunity and self-tolerance. Nat Rev Immunol 1: 126-134.

76. Amigorena S, Savina A (2010) Intracellular mechanisms of antigen cross presentation in dendritic cells. Curr Opin Immunol 22: 109-117.

77. Bachmann MF, Jennings GT (2010) Vaccine delivery: a matter of size, geometry, kinetics and molecular patterns. Nat Rev Immunol 10: 787-796.

78. Akondy R, Monson N, Miller J, Edupuganti S, Teuwen D, et al. (2009) The yellow fever virus vaccine induces a broad and polyfunctional human memory CD8+ T cell response. J Immunol 183: 7919-7930.

79. Goonetilleke N, Moore S, Dally L, Winstone N, Cebere I, et al. (2006) Induction of multifunctional human immunodeficiency virus type 1 (HIV-1)-specific T cells capable of proliferation in healthy subjects by using a prime-boost regimen of DNA- and modified vaccinia virus Ankara-vectored vaccines expressing HIV-1 Gag coupled to CD8+ T-cell epitopes. J Virol 80: 4717-4728.

80. Seder RA, Darrah PA, Roederer M (2008) T-cell quality in memory and protection: implications for vaccine design. Nat Rev Immunol 8: 247-258.

81. Trindade GF, de Santana MG, dos Santos JR, Galler R, Bonaldo MC (2012) Retention of a recombinant GFP protein expressed by the yellow fever 17D virus in the E/NS1 intergenic region in the endoplasmic reticulum. Mem Inst Oswaldo Cruz 107: 262-272.

82. Claser C, Espíndola N, Sasso G, Vaz A, Boscardin S, et al. (2007) Immunologically relevant strain polymorphism in the Amastigote Surface Protein 2 of Trypanosoma cruzi. Microbes Infect 9: 1011-1019.

**Figure 1**. **Characterization of recombinant YF 17D virus expressing *Trypanosoma cruzi* ASP-2_261-380_ fragment.** A) Schematic representation of the ASP-2 protein (Y strain clone 9) presenting the ASP box motifs, the highly conserved VTVxNVxLYNR sequence and the immunogenic fragment comprising amino acids 261 to 380, which includes the CD8^+^ T cell TEWETGQI epitope (modified from and ). B) Diagram showing the corresponding insertion site of ASP-2_261-380_ in the YF genome between E and NS1 genes (indicated by the star). The YF virus genome is translated in a precursor polyprotein which is proteolytically processed by the viral protease complex NS2B NS3 (open arrows), the host signalase (dark arrows), furin (open triangle) and unknown host protease (dark triangle). C) Replication curve in Vero cells of YF17D/ENS1/Tc (full squares), YF17D/NS2B3/Tc (full circles) and YF 17DD (empty circles). Each curve point represents average titer and standard deviation obtained from three different experiments. D) RT-PCR fragments obtained from YF 17DD viral RNA (lane 1); or recombinant YF17D/ENS1/Tc viral RNA collected from the second passage (lane 2) and sixth passage (lane 3). MW (bp), Low Mass DNA Ladder.

**Figure 2**. **Expression of the *T. cruzi* TEWETGQI epitope by the recombinant YF17D/ENS1/Tc virus.** Indirect immunofluorescence assay of Vero cells infected with YF 17DD vaccine virus (A and D), recombinant YF17D/ENS1/Tc viral (B and E), and recombinant YF17D/NS2B3/Tc virus (C and F). Cells were stained with a mouse polyclonal hyperimmune serum to YF 17D (panels A, B and C) or a polyclonal antibodies directed to the TEWETGQI epitope (panels D, E and F). The employed secondary antibodies were labeled with Alexa Fluor 488 (A, B and C) and Alexa Fluor 546 (D, E and F).

**Figure 3** – **Detection of specific antibodies against *T. cruzi* amastigotes after immunization of mice with YF17D/ENS1/Tc or YF17D/NS2B3/Tc** **virus.** Panels A and B show *T. cruzi*-infected Vero cells stained with sera from YF17D/ENS1/Tc virus immunized mice. Panels C and D show *T. cruzi*-infected Vero cells stained with sera from YF17D/NS2B3/Tc virus immunized mice. These preparations were also stained with a FITC conjugated-secondary antiboby (A and C) and DAPI (B and D). Arrows indicate amastigote nests in the cytoplasm of Vero cells. Note the intense serum antibody reactivity (green) with the intracellular parasites. DAPI stained host cell nucleus and also the nucleus and kinetoplast of the parasites. Images obtained from Zeiss Axioplan microscope equiped with epifluorescence (objective 63x).

**Figure 4**. **Trypomastigote-induced mortality and parasitemia in immunized A/J mice.** A) mouse survival after *T. cruzi* challenge. Kaplan-Meier curves of (7 to 12 mice per group) of experimental groups which were immunized with the recombinant viruses YF17D/NS2B3/Tc (●), YF17D/ENS1/Tc (▼), Formulation 1 (50% of YF17D/ENS1/Tc and 50% of YF17D/NS2B3/Tc viruses) (♦) or vaccine YF 17DD control virus (□). Differences between the groups immunized with the recombinant 17D viruses and that one which received the YF 17DD vaccine were statistically significant (****P* < 0.0001, logrank test). B) Average value of trypomastigote-induced parasitemias of immunized A/J mice and intraperitoneally challenged with 250 bloodstream trypomastigotes. The parasitemia for each individual mouse is represented in the graphs for: (C) mice immunized with YF 17DD; (D) mice immunized with YF 17D/NS2B3/Tc; (E) mice immunized with YF 17D/ENS1/Tc; (E) immunized with Formulation 1. The parasitemic peak of mice immunized with Formulation 1 were lower than the parasitemias of mice immunized with YF 17DD or YF17D/NS2B3/Tc (**P* < 0.05, Tukey’s test).

**Figure 5** – **Induction of IFN-γ secreting splenocytes in vaccinated mice before and after *T. cruzi* challenge.** Groups of A/J mice were immunized once or twice with medium (mock) (○), YF 17DD vaccine (□), YF17D/NS2B3/Tc virus (●) and YF17D/ENS1/Tc virus (▼) or alternatively with Formulation 1 (50,000 PFU of YF17D/NS2B3/Tc and 50,000 PFU of YF17D/ENS1/Tc) (♦) and challenged or not with 250 *T. cruzi* trypomastigotes. Spleen cells of each mouse were obtained one week after the first dose (A and C), one week after the second dose (B and D) or two weeks after challenge (E) and after that, were stimulated *in vitro* with YF 17DD inactivated virus (A and B) or TEWETGQI-peptide (C, D and E) to assess cellular responses to YF peptides or to the *T. cruzi* peptide, respectively. Results represent IFN-γ producing cells (SFC) per 10^6^ spleen cells. Asterisks indicate statistically significant differences between groups of immunization in comparison to Mock (A and B) or in comparison to Mock and YF 17DD group (C, D and E) and were done by ANOVA Tukey´s test (****P* < 0.0001; ***P* < 0.01; * *P* < 0.05).

**Table 1**. Immunogenicity of YF 17D recombinant viruses or viral formulations in A/J mice

| **Immunogen** | **Animals (n)** | **Seroconversion (%)** | **PRNT_50_^*^** | |
| --- | --- | --- | --- | --- |
|  |  |  | **GMT ± SD** | **Titer Range** |
| **YF 17DD** | 15 | 100 | ≥ 559 ± 410 | 99 - ≥ 1280 |
| **YF17D/NS2B3/Tc** | 10 | 100 | ≥ 277 ± 465 | 47 - ≥ 1280 |
| **YF17D/ENS1/Tc** | 10 | 100 | 69 ± 76^**^ | 18 - 235 |
| **Formulation 1^a^** | 10 | 90 | 51 ± 25^**^ | 18 - 86 |
| **Formulation 2^b^** | 10 | 50 | 37 ± 26^**^ | ≤ 10 - 82 |
| **Earle’s Medium 199** | 10 | 0 | ≤ 10 | ≤ 10 |

^a^50% of YF17D/ENS1/Tc and 50% of YF17D/NS2B3/Tc viruses

^b^75% of YF17D/ENS1/Tc and 25% of YF17D/NS2B3/Tc viruses

^*^ values indicate the reciprocal of the dilution yielding 50%. PRNT, Plaque Reduction Neutralization Test; GMT, Geometric Mean Titre.

^**^ differences in the titers of neutralizing antibodies virus between YF 17DD and the recombinant viruses or viral formulations were statistically significant (*P* value < 0.05; Kruskal-Wallis test)
